# Impact of the COVID-19 Pandemic and Vaccine Availability on Utilization of Breast Imaging in a Multistate Radiology Practice

**DOI:** 10.1155/2024/6653137

**Published:** 2024-02-09

**Authors:** Andrew K. Hillman, Phil Ramis, Patrick Nielsen, Eric M. Rohren

**Affiliations:** ^1^Baylor College of Medicine, Houston, Texas, USA; ^2^Radiology Partners Research Institute, El Segundo, California, USA

## Abstract

**Method:**

Data were obtained from medical health records across 77 Radiology Partners practices in the US. The data provided us with the total monthly mammography, breast ultrasound, and breast MRI procedures from January 2019 to September 2022. An interrupted time-series (ITS) analysis was conducted to evaluate the effect of the COVID-19 pandemic and the COVID-19 vaccination. We chose March 2020 and December 2020 as critical time points in the pandemic and analyzed trends before and after these dates.

**Results:**

The starting level (at baseline in January 2019) of the total breast imaging procedure volume was estimated at 114,901.5, and this volume appeared to significantly increase every month prior to March 2020 by 4,864.0 (*p* < 0.0001, CI = [3,077.1, 6,650.9]). In March 2020, there appeared to be a significant decrease in volume by 104,446.3 (*p*=0.003, CI = [−172,063.1, −36,829.5]), followed by a significant increase in the monthly trend of service volume (relative to the pre-COVID trend) of 20,660.7 per month (*p*=0.001, CI = [8,828.5, 32,493.0]). In December 2020, there appeared to be a significant decrease in service volume by 69,791.2 (*p*=0.012, CI = [−123,602.6, −15,979.7]). Compared to the period from March to November 2020, there was a decrease in the monthly trend of service volumes per month by 24,213.9 (*p* < 0.0001, CI = [−36,027.6, −12,400.2]). After March 2020, the total service volume increased at the rate of 25,524.7 per month (*p* < 0.0001, CI = [13,828.2, 37,221.2]). In contrast, the service volumes after December 2020 appeared to grow steadily and slowly at a rate of 1,310.8 per month (*p*=0.118, CI = [−348.8, 2970.3]).

**Conclusion:**

Our study revealed that there has been a recovery and a further increase in breast imaging service volumes compared to prepandemic levels. The increase can be best explained by vaccination rollout, reopening of elective/nonemergency healthcare services, insurance coverage expansion, the decline in the US uninsured rate due to government interventions and policies, and the recovery of jobs with employer-provided medical insurance post-pandemic.

## 1. Introduction

The COVID-19 pandemic raised much uncertainty about the future and safe delivery of healthcare. On January 20, 2020, the CDC confirmed its first case of COVID-19 in the United States (US) [1], and by the middle of March 2020, COVID-19 cases had gone up to over 2,700 [2]. Some states in the US began activating shutdown orders and issuing travel advisory [1], and on March 20, 2020, the White House extended all social distancing measures till the end of April 2020 [1].

A survey by the World Health Organization (WHO) showed that countries worldwide reported a significant disruption of about half of essential healthcare services [3]. The United States saw a similar trend as nonessential healthcare services [4, 5], including some breast imaging procedures, were suspended temporarily. A year after the start of the COVID-19 pandemic, a different survey revealed that about 90% of countries still reported one or more disruptions to essential health services. Regardless, there was some recovery of healthcare delivery globally in the first three months of 2021, with only one-third of services disrupted [3].

Some studies have reported significant recovery of overall healthcare delivery to prepandemic levels. Significant, albeit temporary, declines in cancer-related healthcare service utilization have been reported in China shortly following the onset of the COVID-19 pandemic [6]. A single-institution study from Italy [7] identified a delay in women undergoing screening breast imaging procedures, with concern for the potential for an increase in cancer diagnoses in the future. There is, however, limited information on the experience in the US across a wide range of practice locations, particularly in the community setting where much of US healthcare is delivered. Since breast imaging is crucial for the maintenance of health in patients [8], we reviewed the utilization of breast imaging services before, during, and after the COVID-19 pandemic within a large multistate radiology practice to determine the trends in utilization.

## 2. Methods

### 2.1. Source of Data

Data were extracted from the billing data for Radiology Partners, representing 77 practice locations in 35 states of US. Between January 2019 and September 2022, a total of 9,225,254 unique breast imaging encounters were recorded. The data represent a sum of the procedure volumes for breast imaging based on CPT codes (shown in Table 1). Procedure volumes were aggregated by total monthly breast imaging volumes over the 45 months of query. No patient-specific information was included in the data query. This study was approved by the Institutional Review Board of Baylor College of Medicine.

### 2.2. Data Analysis

An interrupted time-series (ITS) analysis was conducted to evaluate the effect of COVID-19 pandemic and the COVID-19 vaccination. It also evaluated the general trend and changes in service volumes from the prepandemic to the post-pandemic period (from January 2019 to September 2022). Our outcome of interest was the total monthly volume of breast imaging services. The ITS focused on two critical time points of the pandemic and their effects on service volumes: initial lockdown in March 2020 and initial vaccination availability in December 2020. We tested for autocorrelation and adjusted for radiologist growth in the practice. Since we only had data on the annual change in radiologists' growth at RP from 2019 to 2022, we assumed that radiologist numbers increased at the same rate per month in a year, depending on the year-specific annual growth increase.

## 3. Results

**Figure 1:**
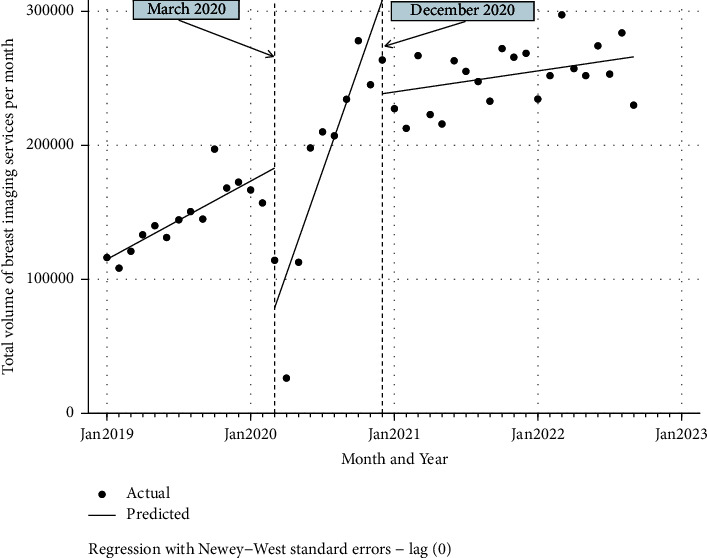
Volumes of breast-imaging procedures between Q1 2019 and Q3 2022, representing aggregate volumes across 10 breast-imaging CPT codes for screening and diagnostic services.

The general trend in breast imaging service volumes from 2019 Q1 to 2022 Q3 is shown in Figure 1. These aggregate volumes represent 10 breast-imaging CPT codes for screening and diagnostic breast-imaging services as shown in Table 2. First, to address the potential impact of practice growth on overall volumes, a simple linear regression analysis was performed. This revealed that radiologist growth at RP during the time of study, January 2019 to September 2022, was not a significant predictor of total monthly service (*p*=0.438). An interaction term between time and radiologist growth was also not a significant predictor (*p*=0.181). Thus, radiologist growth and the interaction term of radiologist growth with time were both excluded from the ITS model.

A Cumby–Huizinga test for autocorrelation revealed autocorrelation was absent in all the lags tested. Therefore, lag was set at lag (0) in the ITS model. The ITS model is specified below.

The starting level (i.e., the baseline level in January 2019) of the total breast-imaging services volume was estimated at 114,901.5, and this volume appeared to significantly increase every month prior to March 2020 by 4,864.0 (*p* < 0.0001, CI = [3,077.1, 6,650.9]). In March 2020, there was a significant decrease in volume by 104,446.3 (*p*=0.003, CI = [−172,063.1, −36,829.5]). This was followed by a period of recovery, with an increase in the monthly exam volumes (relative to the pre-COVID trend) of 20,660.7 per month (*p*=0.001, CI = [8,828.5, 32,493.0]).

Monthly volumes steadily increased until December, 2020, when there was a second significant drop in breast-imaging procedures by 69,791.2 procedures (*p*=0.012, CI = [−123,602.6, −15,979.7]). Although exam volumes recovered after December 2020, the rate of growth of breast-imaging volumes was much lower than after the prior drop in March 2020. As seen in the post-trend output in Table 3, after the first drop in March 2020, the total service volume increased at the rate of 25,524.7 per month (*p* < 0.0001, CI = [13,828.2, 37,221.2]). In contrast, the service volumes after December 2020 increased at a rate of 1,310.8 per month (*p*=0.118, CI = [−348.8, 2970.3]). This difference in monthly exam volume growth (24,213.9 cases per month) was statistically significant (*p* < 0.0001, CI = [−36,027.6, −12,400.2]).

(1) $$\begin{array}{c} Y_{t}{=\beta }_{0}+\beta_{1}\times {\text{time}}_{t}+\beta_{1}\times \left(\text{start}\;\text{of}\;\text{COVID}-19\;\text{in}\;\text{March}\;2020_{t}\right)\\ +\beta_{3}\times \left(\text{time}\;\text{after}\;\text{start}\;\text{of}\;\text{COVID}-19\;\text{in}\;\text{March}\;2020_{t}\right)\\ +\beta_{4}\times \left(\text{vaccination}\;\text{in}\;\text{December}\;2020_{t}\right)\\ +\beta_{5}\times \left(\text{time}\;\text{after}\;\text{vaccination}\;\text{in}\;\text{December}\;2020_{t}\right)+e_{t}. \end{array}$$

## 4. Discussion

In the early days of the COVID-19 pandemic, the US saw a significant drop in elective surgical and medical procedures and services in an effort to mobilize and preserve resources in managing those who had severe COVID-19 symptoms requiring hospitalization and intensive care and those with other medical or surgical emergencies [9, 10].

An analysis of the Census Bureau s Quarterly Services Survey (QSS) data on doctor visits, lab and diagnostic testing, and hospital visits revealed that both Offices of Physicians and Medical Diagnostic Laboratories revenues fell significantly in the second quarter (Q2) of 2020, down 14.9% and 10.9%, respectively, from the first quarter (Q1) of 2020 [9]. Hospital in-patient days dropped significantly by 10.8%, from approximately 54.5 million in Q1 of 2020 to 48.6 million in Q2 of 2020, mainly due to fewer elective surgery procedures at the time [10]. Also, hospital discharges declined by 15.9%, from approximately 9.5 million in the 2020 Q1 to 8.0 million in 2020 Q2 [10]. In most communities in the country, there was a decline in emergency visits by 40% [9].

These findings closely mirror trends seen in our data where we saw massive drop in service volumes by about 104,446 (*p*=0.003, CI = [−172,063.1, −36,829.5]). The finding coincided with the timing of the declaration of national emergency concerning the (COVID-19) pandemic [11] and the subsequent release of recommendations by several medical societies throughout the country. The Centers for Medicare and Medicaid Services (CMS) recommended that nonessential medical and surgical procedures be canceled or postponed [12]. A statement by the Society of Breast Imaging (SBI) on breast imaging during the COVID-19 pandemic in March 2020 recommended that “individual facilities delay screening breast-imaging exams for several weeks or a few months and furthermore diagnostic studies on women without a clinically concerning symptom, such as patients with six-month follow-up, should also be delayed” [13].

On April 13, 2020, the American Society of Breast Surgeons (ASBrS), the National Accreditation Program for Breast Centers (NAPBC), the National Comprehensive Cancer Network (NCCN), the Commission on Cancer (COC) of the American College of Surgeons, and the American College of Radiology (ACR) released a joint recommendation specifically for breast cancer patients without any COVID-related symptoms, requiring some form of breast diagnostic imaging procedure or biopsies. The recommendation served as a guideline for prioritizing, treating, and triaging breast cancer patients during the coronavirus (COVID-19) pandemic. A three-category priority classification for a breast cancer patient was created, depending on COVID impact, patient's condition (including comorbidities), and potential treatment efficacy [14]. In Phase 1 (minimal impact of COVID on hospital resources), procedures prioritized would include patients receiving ongoing therapy, proven recurrence, or certain subtypes of newly diagnosed cancer. In Phase II (increasing COVID impact on resources with limited ICU beds, ventilator capacity, and so on), only urgent procedures would be considered, such as incision and drainage of breast abscesses, evacuation of hematomas, and revascularization procedures following breast surgery. Finally, in Phase III (hospital resources entirely routed to COVID response), only patients with potential life-threatening conditions needing surgical intervention would be considered.

A survey of 77 breast-imaging facilities within the Breast Cancer Surveillance Consortium in the US evaluated the pandemic's impact on clinical practices from March and September 2020 [15]. The study found that almost all (97%) of facilities operated at a reduced capacity at some point during the pandemic. By September 2020, 14% still operated at reduced capacity, even though all facilities were reopened earlier in August 2020. After reopening, 93% of facilities prioritized diagnostic breast imaging over breast cancer screening. Prioritization for both diagnostic and screening imaging services in facilities was based on rescheduling canceled appointments (89% and 96%, respectively), patient demand (84% and 83%, respectively), individual characteristics and risk factors (77% and 73%, respectively), and time since last imaging examination (72% and 71%, respectively). In addition, facilities prioritize diagnostic imaging based on specific indications for diagnostic imaging (89%).

Fewer screening and diagnostic breast-imaging services could negatively impact early breast cancer detection, early recurrence detection, and treatment. Lowry et al. conducted a study on 66 facilities to evaluate the effect of the pandemic on screening and diagnostic breast-imaging cancer detection and biopsy recommendations [16]. The results revealed that there was 24% fewer breast biopsy recommendation with cancer diagnosis from March to September of 2020 compared with the same period in 2019 (1650 recommendations in 2020 vs. 2171 recommendations in 2019, *p* < 0.001). These were mainly due to 38% fewer screen-detected cancers (722 cancers in 2020 vs. 1169 cancers in 2019, *p* < 0.001) versus symptomatic cancers (895 cancers in 2020 vs. 965 cancers in 2019 [7% fewer], *p*=0.27). Asian and Hispanic populations had the most significant decrease in cancer diagnosis, followed by Black women.

The data in this study represent a large, multistate radiology practice of primarily community-based imaging facilities. In comparison to prepandemic trends (from January 2019 but prior to March 2020) where volumes were estimated at 4,864 services per month (*p* < 0.0001, CI = [3,077.1, 6,650.9]), the highest breast-imaging utilization, estimated at about 25,525 per month (*p* < 0.0001, CI = [13,828.2, 37,221.2]), occurred between April and November 2020. We hypothesize this this surge was primarily due to the combination of patients who had to delay receiving services due to the postponement of nonemergency procedures, in addition to those who had originally been scheduled for that time period.

The COVID-19 vaccine rollout in December 2020 ensured a safe return to and reopening of all healthcare services and businesses across the country at near total operational capacity. It has been estimated to have prevented about 18.5 million additional hospitalizations and 3.2 million additional deaths in the US [17]. To ensure reduced transmission and safety of healthcare workers, the federal government issued COVID-19 vaccine mandates in August 2021 [18]. As of January 2022, vaccine mandates were active in 25 states. ACR also released updated recommendations in late 2020 and 2021, ensuring the safe provision of services to patients [19, 20]. The federal government policies and interventions such as the increase in marketplace subsidies following signing of the American Rescue Plan (ARP) Act in March 2021 [21] and the extension of dates for special marketplace special enrollment (federal health exchange) [22] have been crucial in increasing health insurance coverage for many Americans during the pandemic. Also, many workers and their families regained employer-sponsored health insurance coverage as COVID infection levels dropped, vaccination rate increased, and many businesses reopened [23]. It was expected that as more people were vaccinated and received access to health insurance, they were more likely to utilize healthcare services including breast-imaging services. It will likely take years of data to demonstrate a definitive effect, or lack thereof, of COVID-related delays on cancer incidence and mortality compared to prepandemic data [24].

The data from this study show that since January 2021, monthly breast-imaging service volumes across all practice facilities have not fallen below 212,500. We have also seen a slow but steady increase in breast-imaging service volumes of about 1,311 per month (*p*=0.118, CI = [−348.8, 2970.3]). If this remains constant for the rest of 2023, we should expect an estimated additional increase of about 47,200 services by December 2023 compared to that which was reported in December 2020 (*N* = 263,544).

Being a retrospective review, this study has several limitations. First, demographic data on individuals assessing these breast-imaging services were not accessed; therefore, a subpopulation analysis could not be done. Second, although the data represents an aggregate of imaging volumes across 35 states in US, the results were not evaluated on a state-by-state basis, and we recognize that there could be regional heterogeneity of breast-imaging utilization stemming from local factors and policies. Third, the observed impact could be slightly magnified. In order to determine impact on overall breast-imaging volumes, multiple breast-imaging modalities were included in the data query: screening and diagnostic mammography, breast ultrasound, and breast MRI. A single woman may potentially have undergone several of these imaging studies in the course of workup, and a disruption of an earlier study (e.g., screening mammogram) may cascade to follow-up examinations (e.g., ultrasound or MRI).

There is an opportunity for future investigation to further clarify some of these points. Specifically, data could be subsectioned by the geographic region with correlation to the available information on local and regional COVID polices in order to study the role of healthcare policy on access. Data could also be queried by insurance class in order to show the presence or absence of disparities between those classes.

## 5. Conclusion

The COVID-19 pandemic disrupted the flow of healthcare services, including screening and diagnostic breast-imaging services. Contrary to what has been widely reported as a slow recovery of healthcare services compared to prepandemic levels, our observations within a large, multistate practice network reveal that there has been a rebound and a further increase in breast-imaging volumes. The initial increase following the COVID-19 vaccination was most likely due to resuming scheduled breast-imaging services coupled with a surge in services from a backlog of patients. The subsequent increase in services could be explained by broadening the insurance coverage in the US, caused by government interventions and employer-sponsored health insurance, and the reopening of breast-imaging facilities.

## Figures and Tables

**Table 1:** Breast-imaging CPT codes for screening and diagnostic procedures.

Breast imaging services/procedures	CPT codes
Ultrasound, breast, unilateral, real time with image documentation, including axilla when performed; complete	76641
Ultrasound, breast, limited, real time with image documentation, including axilla when performed; limited	76642
Magnetic resonance imaging, breast, without contrast material; unilateral	77046
Magnetic resonance imaging, breast, without contrast material; bilateral	77047
Magnetic resonance imaging, breast, without and with contrast material(s), including computer-aided detection (CAD) (CAD real-time lesion detection, characterization and pharmacokinetic analysis), when performed; unilateral	77048
Magnetic resonance imaging, breast, without and with contrast material(s), including CAD (CAD real-time lesion detection, characterization and pharmacokinetic analysis), when performed; bilateral	77049
Screening digital breast tomosynthesis, bilateral	77063
Diagnostic mammography, including CAD when performed; unilateral	77065
Diagnostic mammography, including CAD when performed; bilateral	77066
Screening mammography, bilateral (two-view study of each breast), including CAD when performed	77067

^*∗*^CAD: computer-aided detection.

**Table 2:** Plot results showing trend and level change estimates.

Parameter	Interpretation	Estimates	Standard error	*p* value	95% CI
*β*_0_	Baseline total service volume in January 2019	114,901.5	4262.4	<0.0001	(106,279.9, 123,523.0)
*β*_1_	Trend per month prior to March 2020	4,864.0	883.4	<0.0001	(3,077.1, 6,650.9)
*β*_2_	Level change in March 2020	−104,446.3	33429.1	0.003	(−17,2063.1, −36,829.5)
*β*_3_	Trend change per month after March 2020 but prior to December 2020 (relative to the pre-March 2020 trend)	20,660.7	5849.8	0.001	(8,828.5, 32,493.0)
*β*_4_	Level change in December 2020	−69,791.2	26603.9	0.012	(−123,602.6, −15,979.7)
*β*_5_	Trend change per month after December 2020 (relative to the March to November 2020 trend)	−24,213.9	5840.6	<0.0001	(−36,027.6, −12,400.2)

**Table 3:** Linear trend estimates after March 2020 and after December 2020.

	Estimates	Standard error	*p* value	95% CI
After March 2020	25,524.7	5,782.7	<0.0001	(13,828.2, 3,7221.2)
After December 2020	1,310.8	820.5	0.118	(−348.8, 2,970.31)

## Data Availability

The authors declare that they had full access to all of the data in this study and the authors take complete responsibility for the integrity of the data and the accuracy of the data analysis. The clinical data used to support the findings of this study are restricted by the Baylor College of Medicine Institutional Review Board in order to protect patient privacy. Data are available from Eric M. Rohren (eric.rohren@bcm.edu) for researchers who meet the criteria for access to confidential data.

## Additional Points

*Summary.* This study evaluated the impact of COVID-19 on breast-imaging service volumes and the recovery of these volumes following the COVID-19 vaccination at a multistate radiology practice group. Our study revealed that there has been a recovery and a further increase in breast-imaging service volumes compared to prepandemic levels. The increase can be best explained by vaccination rollout, reopening of elective/nonemergency healthcare services, insurance coverage expansion, the decline in the US uninsured rate due to government interventions and policies, and the recovery of jobs with employer-provided medical insurance post-pandemic. *Take Home Points*. During the onset and height of the COVID-19 pandemic, there was a sudden and notable decrease in the overall volume of breast-imaging services provided by Radiology Partners (RP) practices. Similar trends were observed in other imaging services offered by various practices and imaging centers throughout the United States. Following the COVID-19 period (up until September 2022), there has been a significant recovery and further increase in breast-imaging services at RP. Initially, the volume of services experienced a sharp increase from March to November 2020, but rates after December 2020 indicate a slower and steady increase in volume growth. The recovery of these services can be attributed to several factors, including the reopening of elective/nonemergency healthcare services, the rollout of vaccinations, and the expansion of health insurance coverage across the United States.
